# High Reproducibility of ELISPOT Counts from Nine Different Laboratories

**DOI:** 10.3390/cells4010021

**Published:** 2015-01-09

**Authors:** Srividya Sundararaman, Alexey Y. Karulin, Tameem Ansari, Nadine BenHamouda, Judith Gottwein, Sreenivas Laxmanan, Steven M. Levine, John T. Loffredo, Stephanie McArdle, Christine Neudoerfl, Diana Roen, Karina Silina, Mackenzie Welch, Paul V. Lehmann

**Affiliations:** 1 Cellular Technology Limited, Shaker Hts. 44122, OH, USA; E-Mails: ayk@immunospot.com (A.Y.K); tameem.ansari@immunospot.com (T.A.); pvl@immunospot.com (P.V.L.); 2 Hôpital Européen Georges Pompidou, Paris 75015, France; E-Mail: nadine.benhamouda@egp.aphp.fr; 3 Copenhagen Hepatitis C Program (CO-HEP), Department of Infectious Diseases and Clinical Research Centre, Copenhagen University Hospital, Hvidovre and Department of International Health, Immunology and Microbiology, Faculty of Health and Medical Sciences, University of Copenhagen, Copenhagen 2650, Denmark; E-Mail: judith@gottwein.eu; 4 Development Translational Medicine, Biogen Idec, Cambridge 02142, MA, USA; E-Mails: srilaxmanan@gmail.com (S.L.); Mackenzie.Welch@biogenidec.com (M.W.); 5 Immuno-Virology Drug Discovery, Bristol-Myers Squibb, Wallingford 06492, CT, USA; E-Mail: steven.m.levine@bms.com; 6 Immuno-Sciences Biology Drug Discovery, Bristol-Myers Squibb, Princeton 08543, NJ, USA; E-Mail: John.Loffredo@bms.com; 7 Nottingham Trent University, Nottingham NG118NS, UK; E-Mail: stephanie.mcardle@ntu.ac.uk; 8 Medizinische Hochschule Hannover 30625, Germany; E-Mail: Neudoerfl.Christine@mh-hannover.de; 9 Pharmasan Labs, Osceola 54020, WI, USA; E-Mail: diana.roen@pharmasan.com; 10 Latvian Biomedical Research and Study Center, Riga LV1067, Latvia; E-Mail: karina@biomed.lu.lv

**Keywords:** ELISPOT, Smart Count™, Log Normal distribution, harmonization

## Abstract

The primary goal of immune monitoring with ELISPOT is to measure the number of T cells, specific for any antigen, accurately and reproducibly between different laboratories. In ELISPOT assays, antigen-specific T cells secrete cytokines, forming spots of different sizes on a membrane with variable background intensities. Due to the subjective nature of judging maximal and minimal spot sizes, different investigators come up with different numbers. This study aims to determine whether statistics-based, automated size-gating can harmonize the number of spot counts calculated between different laboratories. We plated PBMC at four different concentrations, 24 replicates each, in an IFN-γ ELISPOT assay with HCMV pp65 antigen. The ELISPOT plate, and an image file of the plate was counted in nine different laboratories using ImmunoSpot® Analyzers by (A) Basic Count™ relying on subjective counting parameters set by the respective investigators and (B) SmartCount™, an automated counting protocol by the ImmunoSpot® Software that uses statistics-based spot size auto-gating with spot intensity auto-thresholding. The average coefficient of variation (CV) for the mean values between independent laboratories was 26.7% when counting with Basic Count™, and 6.7% when counting with SmartCount™. Our data indicates that SmartCount™ allows harmonization of counting ELISPOT results between different laboratories and investigators.

## 1. Introduction

The T lymphocyte system responds to encounters with viruses such as HCMV via clonal expansion of virus-specific T cells, generating effector and memory cells that can protect the host. The extent of this clonal expansion defines the magnitude of T cell immunity in an individual and is reflected by the frequencies of virus specific T cells in his/her blood. Therefore, one of the primary goals of immune monitoring efforts is to accurately establish the numbers of virus-specific T cells in the blood of infected or vaccinated individuals [1]. Although there is a discrete number of antigen-specific T cells in any blood sample, measuring this number reliably continues to be a major challenge.

Multi center immunoassay proficiency panels reported significant discrepancies in determining the numbers/frequencies of antigen-specific T cells when different laboratories tested the same number of Peripheral Blood Mononuclear Cells (PBMC) from the same donors [2]. The assay results varied more than 100-fold for tetramers, 20-fold for intracytoplasmic cytokine staining (ICS), and 35-fold for ELISPOT. The authors of this report concluded: “The high degree in variability (analyzing the same PBMC samples) makes the comparison between any two labs becomes a game of chance” [2]. 

There are three primary reasons for this high degree of test result variability. The first reason is the lack of standardization of the wet laboratory portion of the assays used for counting antigen-specific T cells [3]. Modifications of the protocol, the use of different test media, or of different assay reagents and instruments, can all fundamentally alter the test results [4,5]. We [6] and others [7] have shown that by standardizing the wet laboratory conditions, the inter-laboratory variability of measuring the frequencies of virus-specific T cells by ELISPOT can be reduced to less than 40%.

The second reason for inter-laboratory discrepancies in measuring frequencies of antigen-specific T cells in PBMC stems from using non-calibrated instruments from different vendors, and the third from subjective data analysis. It has been reported that when different individuals analyze the same flow cytometry data file, the frequencies of antigen-specific T cells varies depending on the expertise of the researcher [8,9]. 

Accurate gating is the primary issue of concern with flow cytometry and with ELISPOT as well. In ELISPOT assays, spots of various sizes and densities are observed, depending on the different quantities and kinetics of cytokines produced by the individual T cells [10]. T cell responses in humans or experimental animals, the primary subjects for immune monitoring by ELISPOT, are rarely clonal. Therefore, we studied antigen-induced CD4 or CD8 cell-derived ELISPOT spot size distribution in humans. .Studies of spot size distributions demonstrated that T cell-derived ELISPOTs closely follow Log Normal Distribution for IFN-γ as well as other cytokines including IL-2, IL-4, IL-5, IL-10, Granzyme B, Perforin, TRAIL, and IL-17 (see Table 1, and the cited references). 

**Table 1:** Reports on Log Normal size distribution of human T cell-derived ELISPOTs.

CD4/CD8 *	Antigen(S)	Analyte(S)	Reference Number
CD4/CD8	Mumps, Dust mite, EBVp	IL2, IL-4, IL-5, IFN-γ	[11]
CD4	PPD	IL-10	[12]
CD4/CD8	Candida, Mumps, PPD, EBVp, Flup, HCMVp	IFN-γ	[13]
CD4	Mumps, EBV	IFN-γ	[14,15,16]
CD8	HIVp	GzB, Perforin, IFN-γ	[17]
CD8	HIVp	TRAIL	[18]
CD4	Vaccinia	IFN-γ	[19]
CD8	HCMVp	IFN-γ	[6]

* Either CD4 or CD8 cells were recalled with the appropriate antigen. When noted “p” represents peptides and all other antigens are inactivated proteins.

To determine whether this notion can be generalized, in a study that (to our knowledge) is the largest of its kind, we analyzed the distributional properties of 334 CD8- and 80 CD4-positive recall responses by PBMC of healthy donors after stimulation with 32 individual viral peptides eliciting CD8 cells and various protein antigens activating the specific CD4 cells [20]. We observed that spot size distributions followed Log Normal distribution, when significance was above 5% using the Kolmogorov-Smirnov test, among all the donors, for all cytokine secretions, and with all antigens that were tested [20]. The notion that T cell-derived ELISPOTs follow Log Normal Distribution has fundamental implications how spot size gates should be set since statistics-based predictions regarding the minimal and maximal spot sizes of a given distribution can be made accurately only when spot sizes follow a Log Normal Distribution. Hence, it should be possible to set gates objectively, thus automatically eliminating count variability resulting from different investigators subjectively selecting counting parameters. 

The aim of this study was to establish the extent to which ELISPOT count variability can be reduced when different laboratories relied on statistics-based automated counting *vs.* the subjective definition of counting parameters. A Reference Plate (RP) with 24 replicate wells for four different cell concentrations resulting in graded spot numbers was sent to nine different participating centers. By scanning this plate on their respective ImmunoSpot® readers and then counting it using fixed parameters, we could measure the extent to which variations between the different instruments contribute to count variability. These laboratories were also provided with an electronic data file of the Reference Plate, the Reference Plate File (RPF) scanned in the reference laboratory. Comparing the counts obtained from the RPF by the different laboratories allowed us to identify differences in counts that are instrument-independent thereby revealing user-dependent count variations only. 

The participating laboratories were asked to analyze the RP and the RPF using three counting modes within the ImmunoSpot® Software: (1) Template Count Mode™, in which the counting parameters are fixed and were provided to all participants by the Reference Laboratory as Reference Counting Parameters. Using these parameters, the count variability should theoretically be zero when the RPF is analyzed. On the other hand, analyzing the RP images, generated on the different instruments in the respective laboratories, while using the Reference Counting Parameters in Template Count Mode™ allowed us to assess the variability introduced by the use of different hardware components of the instruments. In this counting mode, there is no possibility of user-dependent variability. (2) Basic Count Mode™, which gives the user full flexibility to manually tailor the counting parameters according to his/her best judgment for the counting of ELISPOTs. When counting the provided RPF, this mode allows the assessment of user-dependent count variability, with no possibility of instrument-dependent variability. (3) SmartCount™ Mode, which enables users to automatically set counting parameters, including size gates for maximal accuracy and reproducibility of results. While SmartCount™ requires the user to “teach” the software what typical positive and negative wells are, the setting of counting parameters and gates is done automatically by the software based on its statistical analysis of the spots in the positive and negative wells. 

## 2. Experimental Section 

### 2.1. Participants

Nine laboratories with ten different CTL ImmunoSpot^®^ Readers participated in this study. Five participating centers were in Europe: namely Hôpital Européen Georges Pompidou, Paris, France (S5UVM012-00-6536); Copenhagen University Hospital, Hvidore, Hvidovre, Denmark (S5UVM012-00-6519); Nottingham Trent University Nottingham, UK (S5UVM012-00-6509); Medizinische Hochschule Hannover, Hannover, Germany (S5UVM012-00-6518); and Biomedical Research Study Center, Riga, Latvia (S5UV2012-00-6701). Four participating centers were in the USA: namely, Biogen Idec, Cambridge, Massachusetts, USA (S6UNIV-00-7023); Bristol Myers Squibb, Wallingford, Connecticut, USA (S6UNIV-00-7009); Bristol Myers Squibb, Princeton, New Jersey, USA (S6UNIV-00-7011); and Pharmasan Labs, Osceola, Wisconsin, USA (S6ULT-00-8003 & S6ULT-00-8005). All participants were experienced in ELISPOT assay protocols and analysis.

### 2.2. IFN-γ Assay and Reference Plate

Human Interferon-γ ImmunoSpot^®^ Kits (Cat # CTL-H1FNG-1/5M Cellular Technology Limited, OH, USA) were used to perform the ELISPOT assays. The assays were performed according to manufacturer’s instructions. Briefly, the PVDF membranes were coated with capture antibody. The antigen, HCMV peptide pp65 (NLVPMVATV) was dissolved in CTL-Test^™^ Medium (CTL, Cat # CTLT-005) at 2μg/mL and was plated in a final volume of 100 μL per well. The plate containing the antigen was stored at 37 °C in a CO_2_ incubator until the cells were ready for plating. A cryopreserved PBMC sample (20060511F) from an HLA-A2 positive donor with pre-established HCMV pp65 reactivity selected from CTL’s PBMC reference sample library (http://epbmc.immunospot.com) was used for all assays. The PBMC were thawed as described in [21], suspended in CTL-Test^™^ Medium at 2.0 × 10^6^ PBMC/mL, 1.5 × 10^6^ PBMC/mL, 1.0 × 10^6^ PBMC/mL and 0.5 × 10^6^ PBMC/mL; of which 100 μL/well was plated per well, without resting [22], depositing respectively 2.0 × 10^5^ cells/well, 1.5 × 10^5^ cells/well, 1.0 × 10^5^ cells/well and 0.5 × 10^5^ cells/well , each in 24 replicate wells. Plates were incubated for 24h at 37 °C in a CO_2_ incubator. Following development, the plates were air-dried in a laminar flow hood prior to analysis. 

### 2.3. Scanning and Counting, Reference Plate File (RPF)

The RP was scanned at the CTL Reference Laboratory using a CTL ImmunoSpot^®^ S6 UV Ultimate Analyzer (S6ULT-00-9000). This file was provided to the participating laboratories as the RPF. The participating laboratories also scanned the plate on their respective analyzers. General instrument settings could potentially affect count results, such as region of interest, counting window width, camera exposure time, and zoom factor were standardized by CTL among the participants. All data were analyzed with ImmunoSpot® Version 5.0 Software. As described in [23], the ImmunoSpot^®^ Software analyses the digital images, employing sophisticated pattern-recognition software, by utilizing color density in order to distinguish spots from background. After background noise subtraction, the foreground objects are analyzed to identify spots of specific morphology, separating confluent and overlapping spots. Calculation of spot-size distributions is also a built-in function of the ImmunoSpot^®^ software. Data are recorded as Spot Forming Units (SFU) per well. Spot size histograms were created using embedded features of the software.

### 2.4. Statistical Methods

Mean and standard deviations of SFU in the 24 replicate wells were calculated. For individual well data, a boxplot was computed based on the maximum, minimum, and median spot counts from each well. To test the Log-Normal Distribution for spot sizes, Kolmogorov-Smirnov test was used. The normality of spot counts was tested with Shapiro-Wilk methods. Additionally, Q-Q plots were generated for spot-size and spot-counts distributions. In order to analyze counts between different labs, the mean values for each PBMC frequency plated was compared between the participants, and the mean of mean values and standard deviations of the mean values were plotted. The inter-laboratory coefficient of variation (CV) was defined as the percent of standard deviation in the mean value.

## 3. Results and Discussion

### 3.1. The Size of Spots in the Reference ELISPOT Assay Plate Follows Log-Normal Distribution

**Figure 1:**
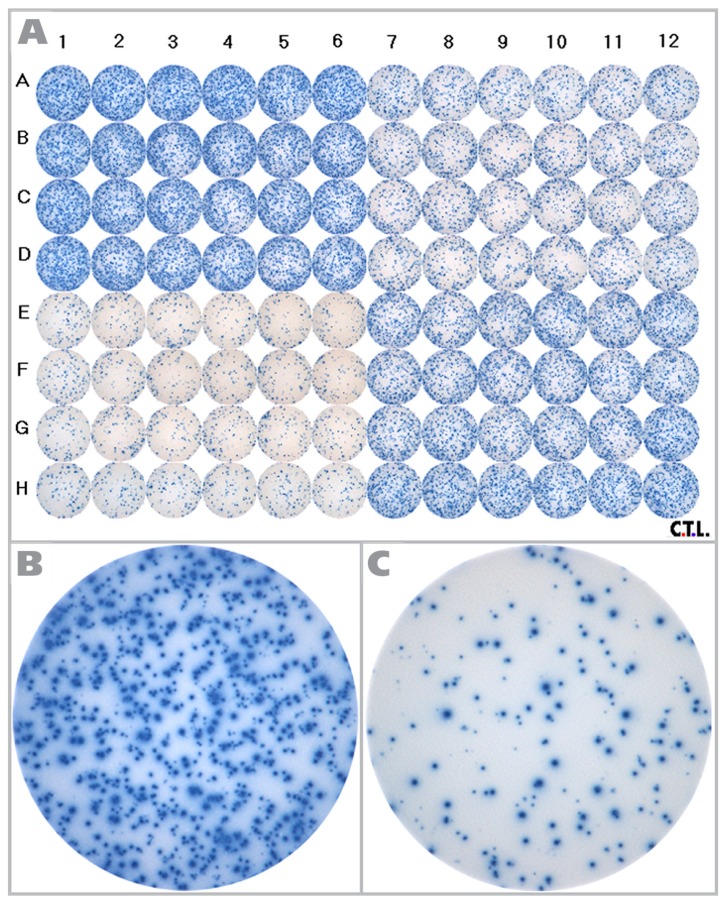
The reference plate used in the study is shown here. (**A**) The Reference Plate was divided into four quadrants. PBMC were plated at 2.0 × 10^5^ (upper left quadrant), 1.5 × 10^5^ (lower right quadrant), 1.0 × 10^5^ (upper right quadrant) and 0.5 × 10^5^ (lower left quadrant) cells per well, with 24 replicate wells each. HCMV pp65 antigen was added to all wells at 2 μg/mL, and after 24h incubation the spots were visualized. Enlarged images are shown for (**B**) 2.0 × 10^5^ cells/well and (**C**) 0.5 × 10^5^ cells/well.

The RPF is shown in Figure 1, along with representative wells for the different cell densities tested. As can be seen in the enlarged well images (Figure 1B,C), a wide range of spot sizes are present. 

**Figure 2:**
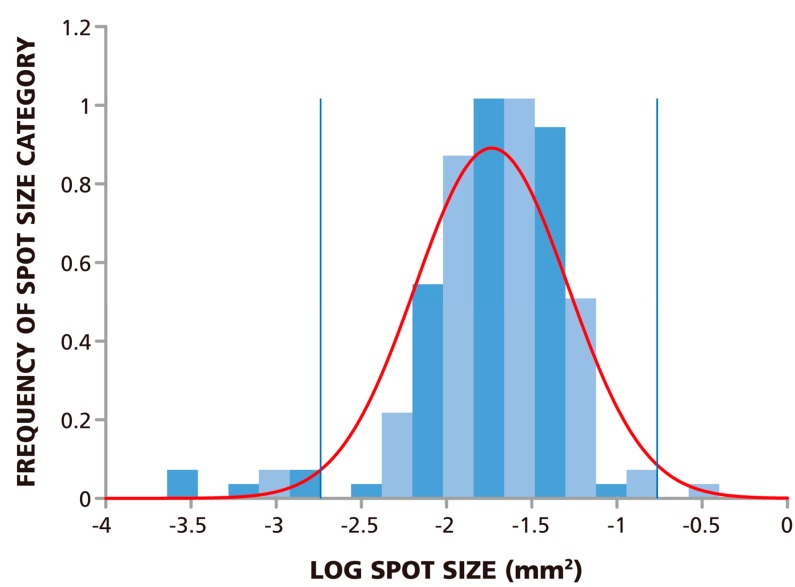
ELISPOT sizes on Reference Plate follow Log Normal Distribution. The experimental size distribution of 148 ELISPOTs on the Reference Plate was established by sampling replicate wells with 1.5 × 10^5^ cells plated per well. The normality of the spot size distribution was tested using Kolmogorov-Smirnov goodness of fit test which resulted in a *p*-value of 0.146 for a target significance level of α = 0.05. The fitted Log Normal curve is overlaid in red, and the lower and upper spot size gates calculated from this curve with >99% confidence are shown by the blue lines.

Since the spots are of different sizes, determining the cut-off value for the smallest and largest spot size to be counted poses a challenge even to ELISPOT experienced investigators, and this judgment call will inherently be subjective. If these spots, like ELISPOTs in general, also follow Log Normal size distribution, statistics-based algorithms can be used to set the lower and upper gate. We therefore studied the size distribution of the spots in the RP. We analyzed 148 spots and found that the measured spot size distribution approximated Log Normal with a *p*-value of 0.146, at a significance level of 0.05 via the Kolmogorov-Smirnov test. In this test, the p value needs to be larger than the target significance level to be significant. The size distribution for spots at 1.5 × 10^5^ PBMC per well are shown in Figure 2 along with a fitted Log Normal curve. This Log Normal size distribution justifies the use of the SmartCount™ feature of the ImmunoSpot® Software, which based on parametric statistics, determines the upper and lower spot-size gates with 99% confidence. The gates calculated by the software for the example provided in Figure 2 are shown by blue lines. Spots that exceed the upper gate are defined as originating from cell clusters and the software automatically calculates the number of spots (*i.e.*, analyte secreting cells) it would take to generate such a cluster. Spots that are smaller than the lower limit are not included in the spot counts.

### 3.2. Reproducibility of Spot Counts for each Well within Repeat Scans

**Figure 3:**
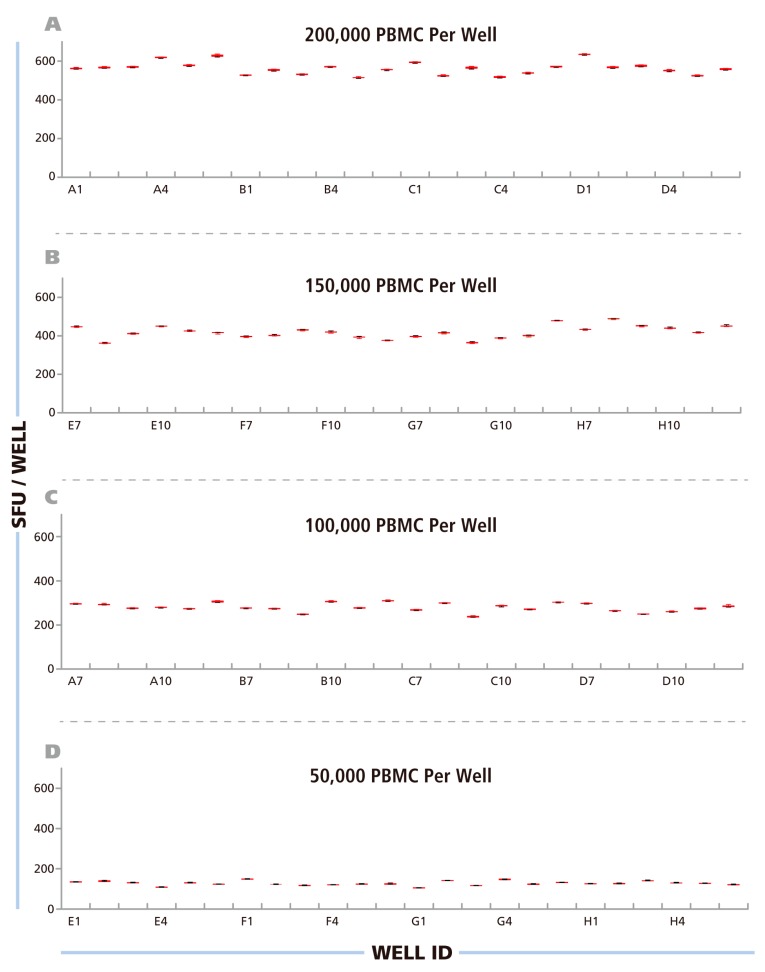
Repetitive scans show minimal intra-well variability of ELISPOT counts – inter-well variability for replicates follows Normal distribution. The Reference Plate was scanned 80 times consecutively over a period of 7 hours. The resulting image files were counted with fixed Reference Counting Parameters. For each well, for the four Reference Plate quadrants (**A**) at 200000 PBMC /well, (**B**) 150000 PBMC/well, (**C**) 100000 PBMC/well and (**D**) 50000 PBMC/well, the mean of the 80 scans is shown with SD. Note, that error bars are <1% and therefore barely visible in the graphs.

After having established, as detailed above, the upper and lower gates for counting the RP, and saved these as the Reference Counting Parameters for this study, we proceeded to systematically address the issue of instrument-dependent count reproducibility. Using an ImmunoSpot® Reader in the Reference laboratory, we scanned the RP eighty successive times, from immediately after powering up the machine through 7 h of continuous use. In this way, we intended to establish the scan-to-scan consistency of the well images, and whether either a warm-up period or overheating of the machine would influence the results. The images obtained through these 80 scans were counted using the Reference Counting Parameters. Mean spot counts and standard deviations for the 80 scans are shown, as box plots, for each well in Figure 3 with wells containing the same numbers of PBMC shown in different panels (A–D). The CV for the spot counts for each well was <1%, barely visible in the graphs. The data establishes a high scan-to-scan consistency for the image files that is not affected by warm up time or by instrument overheating. The data also shows that the spots on the RP did not measurably fade during the 80 repeat scans. This finding was important for our subsequent study, sending the RP to different laboratories for successive scanning and comparisons of counts.

### 3.3. ELISPOT Counts for Replicate Wells Show Normal Distribution

The data in Figure 3 A–D also show that the major variation in spot counts among replicate wells result from actual assay result variations, rather than the instrument counting individual wells differently. When the PBMC were plated at 2 × 10^5^ cells per well, for example (Figure 3A), the mean spot counts for the 24 replicate wells was 539 SFU/well, but the counts for the individual wells ranged from 487 SFU/well to 605 SFU/well. This type of variation can be expected because relatively few antigen-specific T cells (in this case around 539) are contained within the 2 × 10^5^ PBMC plated in 100 μL, and the exact number of antigen specific cells will underlie sampling-dependent well-to-well variations. To prove that the nature of this variation is random, the data in Figure 3A–D were subject to statistical analysis of the distributional properties of the spot counts. For each condition, the distributional properties of the spot counts were analyzed in the 24 replicate wells using the Shapiro-Wilk normality tests with a significance level of α = 0.05. For all four cell concentrations, the spot count variations within the replicates fit Normal Distribution, with p values greater than the target significance level (0.811, 0.416, 0.144, and 0.358, respectively). 

The Normal Distribution of ELISPOT counts among replicate wells on the RP confirms what we previously reported using IFN-γ transfected CHO cells [24], and was also observed involving higher numbers of replicate wells, testing primary PBMC for peptide-induced recall responses of CD8 cells [25]. Therefore, parametric statistical analysis, including the use of the Students’ t-test is suitable for comparing different experimental conditions of ELISPOT tests, such as identifying positive antigen-induced spot counts over the medium control. The Normal Distribution of ELISPOT counts among replicate wells also allows for accurate predictions regarding the numbers of replicate wells that are needed to statistically show a significant difference between given experimental conditions. 

### 3.4. Linear Relationship between PBMC Numbers Plated per Well and Spot Counts per Well

**Figure 4:**
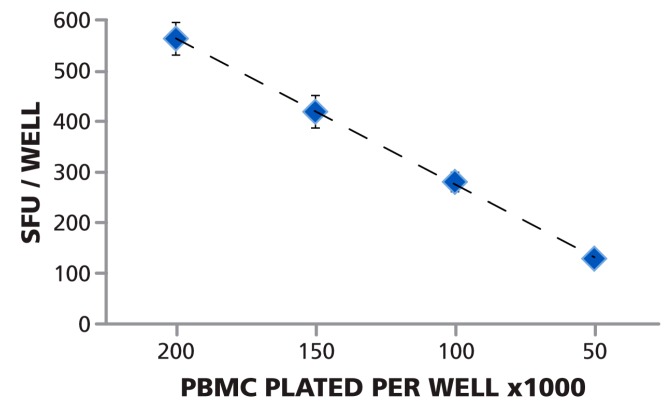
Spot counts and number of cells plated follow a linear function. The Reference Plate contained PBMC plated at 2.0 × 10^5^, 1.5 × 10^5^, 1.0 × 10^5^, and 0.5 × 10^5^ cells per well, 24 replicates of each. The plate was scanned, and counted with the Reference Counting Parameters. The mean of means (of the 24 replicates in 80 scans) and the corresponding SD is shown for the four cell numbers plated. The data approximates ideal linearity with R^2^ = 0.9985.

As shown in Figure 4, the number of PBMC plated per well, and the measured mean spot count per well (calculated from the 24 replicates for each PBMC number) followed a linear function. Since ELISPOT assays measured cytokine secretion by individual antigen-specific T cells, a linear relationship can be expected provided that the cells form a monolayer on the membrane, and access of the antigen-specific T cells to antigen-presenting cells is not limiting. When directly visualizing cells in a 96 well plate, 0.5 × 10^5^ PBMC per well is the lower limit for a confluent cell lawn (data not shown). The data also show that ImmunoSpot^®^ software recognized spots correctly even if they occurred in different densities (including confluent spots) and over different background coloration. As shown in Figure 1B, at 2.0 × 10^5^ PBMC per well, spots are beginning to crowd and excess cytokine leads to a moderate blue coloration of the background due to an ELISA effect. As a consequence, the background color density at 2.0 × 10^5^ cells per well is higher than that of faint spots at 0.5 × 10^5^ cells per well (Figure 1C). For precise spot recognition and counting, the software needs to automatically compensate for such changes in background. The linearity between the automated spot count and cell numbers plated shows that automatic spot recognition features of the ImmunoSpot^®^ Software compensated for varying background coloration and spot crowding, providing the correct count at all four spot densities. 

All the above data, obtained with a single instrument in a single laboratory, set the basis for the subsequent comparisons among the nine participating laboratories using different instruments and involving independent investigators while analyzing the same physical RP or a single electronic file of it, RPF. 

### 3.5. Variation of ELISPOT Counts due to Image Acquisition-Based Differences of Instruments

When the same image file is analyzed on different instruments using the same software and software parameters, one would expect to obtain the same results. The RPF was sent to the nine participating centers, along with Reference Counting Parameters that provided fixed values for all variables of counting; including spot separation, spot size gating, background balance, and more. When the RPF was counted using these Reference Counting Parameters in the ImmunoSpot^®^ Software’s Template Count™ Mode by the respective laboratories, the counts were identical for all nine participating laboratories, with a SD of counts for each PBMC number plated, obtained among the different laboratories being 0 (data not shown). As expected, when software-based variations are excluded, the counting of the same files with a fixed template leads to identical results in different laboratories.

**Figure 5:**
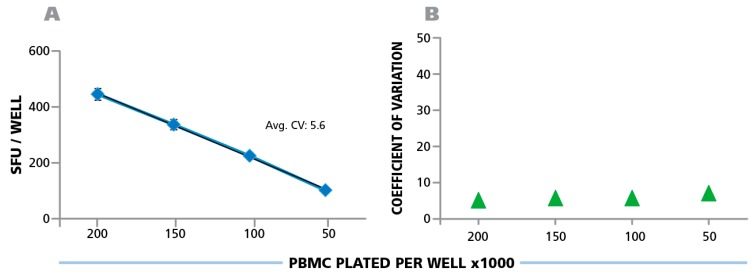
Hardware-dependent variability of ELISPOT counts generated on different ImmunoSpot® Readers. The physical Reference Plate was sent to the nine participating laboratories for scanning, one of which had two instruments. The well images obtained with the 10 different instruments were counted using the fixed parameter set provided by the Reference Laboratory, the Reference Counting Parameters implemented in Template Count™ Mode. (**A**) The mean (from 10 analyzers) of mean (from 24 replicates of each PBMC concentration) and the corresponding SD is shown with the average CV for the entire plate, (**B**) the CV is shown for the four cell densities.

Counting ELISPOTs from the RP scans (that were generated on the different instruments) with fixed parameters establishes the degree of contribution of instrument hardware to count variability. A number of variables, such as camera settings, illumination, precision of stage movement, and more, can individually contribute to differences in the well images obtained. To test for variability caused by the hardware components, the RP was shipped to each participant, scanned at the laboratory’s respective ImmunoSpot® instrument, and then shipped to the next laboratory. Subsequently, the electronic files generated by each laboratory were analyzed using the Reference Counting Parameters. The mean count for each analyzer, among their respective 24 replicates, was first computed. Then, the mean (from 10 analyzers) of the mean (of 24 replicates per cell density) and the corresponding SD of mean counts (from 24 replicate values) obtained on the 10 different instruments in the 9 laboratories is shown in Figure 5A. The average CV for the entire plate was 5.6%, the CV for the different cell densities is shown in Figure 5B; and was highest at 0.5 × 10^5^ PBMC per well at 6.7%. The inter-well CVs between the 24 replicates for the 10 analyzers were 33.0 ± 2.3 for 2.0 × 10^6^ cells/well, 32.4 ± 0.7 for 1.5 × 10^6^ cells/well, 21.1 ± 1.7 for 1.0 × 10^6^ cells/well, and 10.3 ± 0.8 for 0.5 × 10^6^ cells/well. This variation is due to inherent assay variations (Figure 3 and Figure 7). The data established that the hardware of the different ImmunoSpot^®^ analyzers provided image consistency leading to less than 7% count variation. It should be noted that the analyzers involved in this study were standard production items that had not been especially harmonized to each other. Such additional harmonization of instruments further reduces the instrument-to-instrument variability, and can be done on special request e.g., for analyzers participating in a clinical study. 

### 3.6. Automated Counting as Opposed to Subjective Counting by Different Investigators Results in Similar Counts

Having established the contribution of assay-inherent variability (spot size distribution and variation within replicate wells), we established the extent to which user judgment affects ELISPOT counts. In particular, we were interested in comparing counts generated using the statistics-based size gating in SmartCount™ mode of the ImmunoSpot® Software (see Figure 2) with the Basic Count™ mode in which all counting parameters can be independently set by the user according to their best judgment. SmartCount™ fundamentally differs from Template Count™ as all parameters are pre-assigned and fixed in the latter. In the SmartCount™ mode sensitivity is set automatically for each well, but the user needs to initiate auto-gating by selecting typical wells (to which there is a subjective element). Based on the images selected the software will automatically analyze spot size distributions, and set the upper and lower spot size gate for counting. Thus, all the parameters that the user is required to adjust manually in Basic Count™ are automatically fine-tuned by the software in SmartCount™.

**Figure 6:**
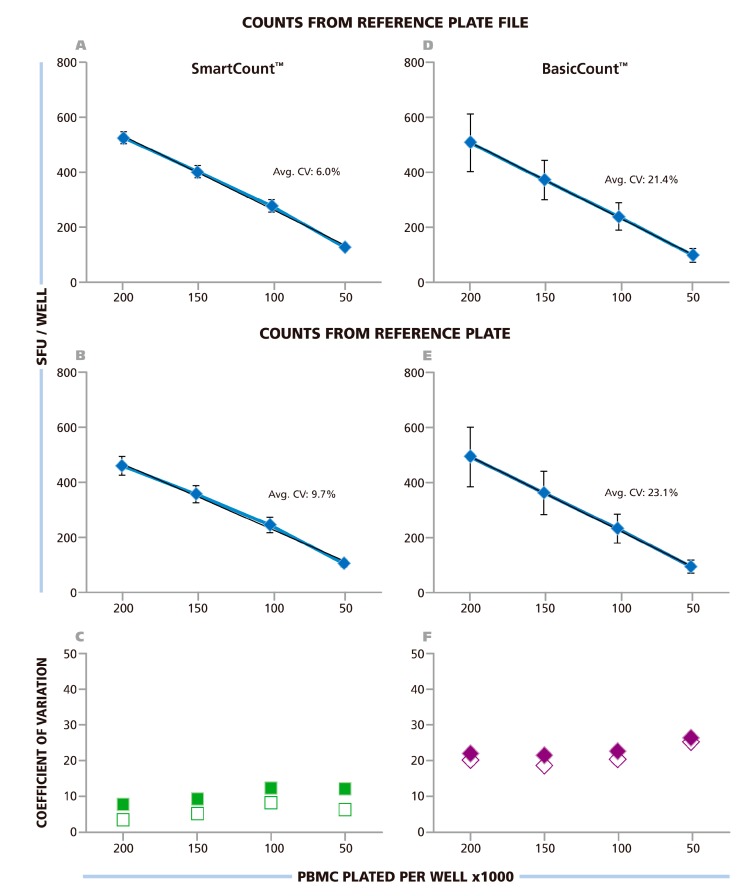
Spot counts from ten labs using SmartCount™ or Basic Count™ respectively. The Reference Plate File (**A** & **D**) or the Reference Plate scanned with the 10 different instruments of the nine participating laboratories (**B** & **E**) were counted either in SmartCount™ mode (**A** & **B**), or in Basic Count™ mode (**D** & **E**). In SmartCount™ mode, the investigators were permitted to pick any well(s) of the Reference Plate as the representative well(s) as long as a total of 500 cumulative spots, from replicate wells were made available for the software to apply statistical analysis. In Basic Count™ mode, the investigators fine-tuned counting parameters manually to accomplish the count according to their best judgment. (**A**–**D**) The mean (from 10 laboratories) of mean (from 24 replicates) and corresponding SD is shown in these panels with the average CV for the entire plate specified. (**C** and **F**) The CV for the four cell densities are shown with the counts for the Reference Plate File in open symbols, and from the Reference Plate in closed symbols, as specified.

For setting automatically size gates in SmartCount™ mode, the investigators were allowed to select any of the wells on the RPF as “representative wells”. It should be noted that the wells differed in spot density and in background coloration (Figure 1). Each of the nine investigators counted the RPF in SmartCount™ mode. This approach established count variability independent of analyzer hardware, since each counted the same RPF, and of subjective manual parameter setup. Only the selection of the representative wells was subjective. The mean (from the ten analyzers) of mean (from 24 replicate wells) value and corresponding SD for the ten independent counts are shown in Figure 6A. The average CV for the entire plate was 6%, the CV for the replicate wells with the different cell densities is shown in Figure 6C being highest at 8.2% with 1.0 × 10^5^ cells per well. The data show that spot counts generated by the different investigators using SmartCount™ displayed lower than 8.2% deviation, even though these investigators were permitted to select representative wells subjectively from the 96 wells of the plate, and even though these wells contained four different levels of spot densities and background coloration.

In the next step, SmartCount™ was used as above, however, the nine investigators counted the image files generated by scanning the RP on their respective instruments. In this setting, the variability of the analyzers adds to the variability of counts generated by implementing SmartCount™. Means of means (from the ten analyzers and 24 replicate wells) and SD for these ten independent counts are shown in Figure 6B. The average CV for the entire plate was 9.7%. The CV for the different cell densities is shown in Figure 6C, and was highest at 11.5%, with 1.0 × 10^5^ cells per well. Thus, the spot counts generated by the different investigators on their respective instruments were lower than 11.5% different when they used SmartCount™ with random selection of representative wells. 

The investigators subsequently used the Basic Count™ mode of the ImmunoSpot^®^ Software to analyze the RPF, and the file they generated from the physical RP using their respective analyzers. The Basic Count™ mode of the ImmunoSpot^®^ Software permits users to fine-tune any and all of the counting parameters using their best judgment for optimal counting including manual size gating. The nine participants of this trial, all of whom had extensive training and first-hand experience with ELISPOT analysis, we asked to set the parameters they thought would most precisely count the RP. The results for counting the RPF in Basic Count™ mode are shown in Figure 6D, and those from counting the RP images obtained on their own reader, in Figure 6E. The average CV for the spot counts obtained from the RPF was 21.4%, and for scans of the RP on the different instruments it was 23.1%. The CV for the different cell densities is shown in Figure 6F, reaching up to 25% at 5.0 × 10^5^ cells per well for the RP. Thus, the variability of spot counts was more than 3-fold higher when the counting parameters were established by experts according to their best judgment, as compared to the automated adjustments made using SmartCount™. 

### 3.7. Average of Subjective Counts Approximates that of SmartCount™

**Figure 7:**
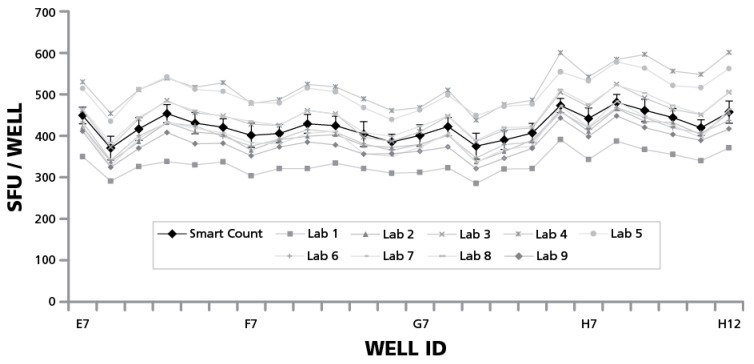
Average of subjective counts approximates that of SmartCount™. Spot counts obtained by the nine participating scientists in Basic Count™ mode are represented by grey lines with different symbols, as specified. The average counts obtained in SmartCount™ mode by the nine scientists and the respective SD for each well ID are shown with the black line. Data are shown for the 1.5 × 10^5^ cells/well quadrant of the RP, with the well IDs specified. The data are representative for the other three quadrants of the plate as well.

The above data showed that the participating expert scientists would generate substantially different counts when analyzing to their best judgment in the Basic Count™ mode, as compared to the automated parameter setup in SmartCount™ mode. To better understand the reason for this discrepancy, we compared the Basic Count™ data provided by the individual laboratories with the counts obtained using SmartCount™. In both cases, the same image material, the RPF, was analyzed. As can be seen in Figure 7, some of the scientists came up with higher subjective counts using Basic Count™ when compared to SmartCount™, and some with lower, but the mean of the nine subjective counts approximated the count generated based on the automated parameter setup of SmartCount™. These results validate the accuracy of the counts generated by SmartCount™. 

### 3.8. Discussion 

The immune monitoring world has been alarmed by a proficiency panel report which made the observation that when analyzing the same PBMC samples, different labs provide different results for T cell assays leading to any comparison between them meaningless [2]. In follow-up studies, the same panel found that when different individuals analyze the same flow cytometry data file, *i.e.* when experimental variation of data is eliminated and only the evaluation of data is being tested, the frequencies of antigen-specific T cells still varies alarmingly. This finding initiated the formation of expert panels with the goal of finding ways to standardize flow cytometry data analysis [26]. Work in progress by the same panels suggests disturbing differences in ELISPOT counts when different individuals analyze the data, relying on subjective judgment, and using instrumentation and software provided by various vendors [5].

One way of reconciling discrepancies is involving many expert judgments, and to accept the consensus as the correct result. Notably in our study, the individual counts differed significantly from each other when the nine experts used their best judgment to analyze a RPF (Figure 6D). The average count, *i.e.* the “consensus correct count”, however, matched the counts that the automated parameter settings in SmartCount™ generated.

The alternative, and in our opinion more reliable, way of establishing correct spot numbers is by understanding the basic scientific principles that underlie T cell ELISPOT assays, and their analysis. We first engaged in this quest around 15 years ago, analyzing the ELISPOT signature of individual T cell clones [27]. When these T cells were seeded in a monolayer of antigen presenting cells (EBV-transformed autologous B cell clones), spots were seen that covered a wide range of spot sizes. The analysis of the spot size distribution, however, showed a perfect bell shaped curve, consistent with normally distributed spots. When the spots were counted based on the assumption of Log Normal distribution, the numbers of T cells plated exactly matched the numbers of ELISPOTs detected [27]. Interestingly, the spot sizes and densities were dependent on the antigen dose used for stimulating the clone, and when the clone was last re-stimulated, but in each case the spot sizes followed Log Normal distribution. If spots follow a Log Normal distribution, one can tell with very high certainty which spots belong to that distribution. Spots that are larger than 3 SD of the mean spot size result with 99.73% likelihood from cell clustering, and should not be counted as single events, but rather the number of spots that would take to create a cluster of that size should be estimated. Clustering can also be experimentally verified by seeding decreasing numbers of T cells, in which case the chance for random clustering exponentially decreases. The number of truly oversized spots that originate from individual cells will proportionally decrease with the numbers of T cells plated, and oversized spots resulting from clusters, however, will become rare and disappear as the cells are diluted. Spots that are smaller than 3 SD of the mean spot size do not belong to the same Log Normally distributed population, and can be gated out with 99.73% certainty. Therefore, when spots show Log Normal distribution, as was also seen for the RP (Figure 2), one can use an objective statistical approach to set gates for counting. In contrast, as it will become obvious when closely studying Figure 1B,C, it is almost impossible for a human to reliably create a cut-off for counting smaller spots, and different individuals will make different judgment calls. This is why the data generated by SmartCount™ by different investigators is very similar; whereas those obtained by subjectively fine-tuning parameters in Basic Count™ mode is not (Figure 6A *vs.* Figure 6D). 

For all antigens we tested so far, and for all T cell cytokines measured, therefore, spot sizes were distributed in bell shaped curves consistent with following Log Normal distribution. In general, CD4 cells produce more cytokine, on a per cell basis, than CD8 cells do, but in each case the spot sizes follow Log Normal distribution [19,28]. Recently activated effector/memory T cells produce more cytokine on a per cell basis than memory cells that had been primed in the distant past, but in both cases the spot sizes approximate Log Normal distribution [14]. Therefore, the mean spot sizes may vary dependent on the CD4 or CD8 cell type, on the antigen history, or the antigen dose/affinity of the T cell response, but the size distribution apparently does not. Short peptides (shorter than 11 amino acids) recall CD8 responses and entire proteins recall CD4 responses. Longer peptides (15 amino acids or more) may recall responses from CD4, CD8, or both cell types. When both cell types become activated, spot sizes can exhibit bi-modal spot curves, each following Log Normal distribution. Similarly, polyclonal stimulation with Phytohemagglutinin (PHA) elicits responses from CD4, CD8, and cells from the innate immune system leading to tri-modal spot curves, each one exhibiting Log Normal distribution. Therefore, biologically mixed responses should take these complex spot curves into account. 

Starting with Version 5.4, the ImmunoSpot® Software has a built in feature that permits automated testing of whether an experimental ELISPOT result matches Log Normal distribution, and whether gates established on the basis of the Log Normal assumption for one test subject/ antigen combination also apply for other test subjects, and permits automated correction, if required. As far as we can tell, at this time, those are rare exceptions with uni-modal Log Normal distribution being the rule. 

## 4. Conclusions 

The Log Normal size distribution of Spot Forming Unites in T cell ELISPOT assays allows us to make statistics-based accurate predictions regarding the smallest and largest spot sizes to be included in counting. Using this automated statistics-based approach, SmartCount™, the counts obtained from ten ImmunoSpot® Analyzers in nine different laboratories were 6.7% apart. While the subjective counts generated by the nine investigators deferred by 26.7%, the mean of these counts matched the SmartCount™ result. This multi-center study, therefore, showed that ELISPOT count harmonization among different laboratories is feasible providing scientifically validated, objective counts. 

## Abbreviations

RP: Reference Plate: The physical plate sent to each investigator to be scanned and counted
RPF: Reference Plate File: A scanned image of the Reference Plate, scanned at the Reference laboratory, and sent to each investigator to be counted only
PBMC: Peripheral Blood Mononuclear Cells
HCMV pp65: Peptide from the human cytomegalovirus antigen
SFU: Spot Forming Unit: Readout of an ELISPOT assay on the plate membrane
